# Adipose Mesenchymal Stem Cell Secretome Modulated in Hypoxia for Remodeling of Radiation-Induced Salivary Gland Damage

**DOI:** 10.1371/journal.pone.0141862

**Published:** 2015-11-03

**Authors:** Hye-Young An, Hyun-Soo Shin, Jeong-Seok Choi, Hun Jung Kim, Jae-Yol Lim, Young-Mo Kim

**Affiliations:** 1 Department of Otorhinolaryngology-Head and Neck Surgery, Inha University College of Medicine, Incheon, Republic of Korea; 2 Translational Research Center, Inha University College of Medicine, Incheon, Republic of Korea; 3 Department of Radiation Oncology, Inha University College of Medicine, Incheon, Republic of Korea; University of Torino, ITALY

## Abstract

**Background and Purpose:**

This study was conducted to determine whether a secretome from mesenchymal stem cells (MSC) modulated by hypoxic conditions to contain therapeutic factors contributes to salivary gland (SG) tissue remodeling and has the potential to improve irradiation (IR)-induced salivary hypofunction in a mouse model.

**Materials and Methods:**

Human adipose mesenchymal stem cells (hAdMSC) were isolated, expanded, and exposed to hypoxic conditions (O_2_ < 5%). The hypoxia-conditioned medium was then filtered to a high molecular weight fraction and prepared as a hAdMSC secretome. The hAdMSC secretome was subsequently infused into the tail vein of C3H mice immediately after local IR once a day for seven consecutive days. The control group received equal volume (500 μL) of vehicle (PBS) only. SG function and structural tissue remodeling by the hAdMSC secretome were investigated. Human parotid epithelial cells (HPEC) were obtained, expanded *in vitro*, and then irradiated and treated with either the hypoxia-conditioned medium or a normoxic control medium. Cell proliferation and IR-induced cell death were examined to determine the mechanism by which the hAdMSC secretome exerted its effects.

**Results:**

The conditioned hAdMSC secretome contained high levels of GM-CSF, VEGF, IL-6, and IGF-1. Repeated systemic infusion with the hAdMSC secretome resulted in improved salivation capacity and increased levels of salivary proteins, including amylase and EGF, relative to the PBS group. The microscopic structural integrity of SG was maintained and salivary epithelial (AQP-5), endothelial (CD31), myoepithelial (α-SMA) and SG progenitor cells (c-Kit) were successfully protected from radiation damage and remodeled. The hAdMSC secretome strongly induced proliferation of HPEC and led to a significant decrease in cell death *in vivo* and *in vitro*. Moreover, the anti-apoptotic effects of the hAdMSC secretome were found to be promoted after hypoxia-preconditioning relative to normoxia-cultured hAdMSC secretome.

**Conclusion:**

These results show that the hAdMSC secretome from hypoxic-conditioned medium may provide radioprotection and tissue remodeling via release of paracrine mediators.

## Introduction

New irradiation (IR)-induced salivary hypofunction occurs in 30,000–50,000 patients/year in the United States. Salivary hypofunction, which is the most common long-term complication of radiotherapy,[1] usually occurs shortly after treatment and leads to an irreversible, life-long problem.[2] Furthermore, the additional cost of care is a great burden on xerostomic patients, which suggests a need for development of new pharmaceutical treatments.[3,4]

Ionizing radiation leads to direct acute damage to normal tissue by disruption of the atomic structure. Ionizing radiation also leads to synthesis of reactive oxygen species (ROS) by radiolysis of water, which can cause indirect oxidative tissue damage.[5] Indirect tissue injury can also occur via the proinflammatory cytokine/chemokine cascade, leading to chronic inflammation and fibrosis. At that time, stem cells may play an important role in the repair and regeneration of damaged tissue. Depletion of stem cells causes failure to replenish the parenchymal and endothelial cell loss. However, there are no satisfactory therapies to restore IR-induced salivary gland (SG) damage; accordingly, studies to develop new biological insights for emerging treatments such as stem cell therapy are warranted.[6]

Regeneration of IR-damaged SGs necessitates considerable repopulation of salivary epithelial, endothelial, and resident SG stem/progenitor cells. SG stem/progenitor cells are capable of readily engrafting into damaged SGs and differentiating into functioning SG cells. Intraglandular transplantation of c-Kit-positive SG progenitor cells has been shown to restore SG tissue homeostasis and function following radiation damage.[7,8] However, the promising therapeutic effects of mesenchymal stem cells (MSC) seem unlikely to involve the ability of multipotent differentiation. Animal studies using bone marrow-derived mononuclear cells and MSC from bone marrow or adipose tissue have shown favorable results, but these studies all used heterogeneous cell populations isolated by conventional methods, which exclude general conclusions.[9–12] We recently attempted to determine whether single cell-derived homogeneous MSC could survive and differentiate into SG epithelial cells in recipient SGs.[13] In our experiments, clonal MSC directly transplanted into SGs contributed to improvement of SG damage without long-term engraftment. Another recent study revealed that systemically infused MSC restored SG hypofunction, but a low number of MSC were found to transdifferentiate into SG epithelial cells.[14] These results suggest that a paracrine effect rather than transdifferentiation accounts for the regeneration of radiation damage by promoting the survival of endogenous stem/progenitor cells or neovascularization.

Many studies have emphasized the role of immunomodulation and trophic effects rather than the differentiation paradigm of stem cells, in which tissue injury stimulates stem cells to secrete many trophic factors that are together defined as the MSC secretome.[15] Secretomes are released from transplanted or homing MSC from other tissues via activation by cross-talk with injured cells or inflammatory microenvironments. To improve the function of secretomes, MSC could be modulated by physiological (hypoxic or low pH) or molecular preconditioning and/or genetic modifications prior to transplantation.[16,17] IR-damaged tissue exhibits a hostile microenvironment owing to oxidative tissue damage, ischemia, inflammation, and fibrosis. We speculate that MSC activated by hypoxic conditions could be enhanced to improve cell survival against harsh microenvironments and may exert more beneficial effects on IR-damaged SG than MSC from the inactive state. In this study, we cultured human adipose mesenchymal stem cells (hAdMSC) under hypoxic conditions and performed secretome analysis. We subsequently investigated whether systemic infusion of the conditioned hAdMSC secretome could involve tissue remodeling of IR-damaged SGs and restore salivary hypofunction. In addition, we addressed the therapeutic mechanisms of the MSC secretome as a candidate therapeutic tool.

## Materials and Methods

### Ethics Statement

This study was approved by the Animal Ethics Committee of Inha University Hospital (Permit Number: 110707–02). Animals were cared for according to established institutional guidelines, and all efforts were made to minimize suffering. Surgeries were performed under anesthesia after xylazine (10 mg/kg) premedication and intraperitoneal injection of ketamine (110 mg/kg).

### Preparation of hAdMSC secretome

The MSC used in this study were derived from surplus frozen cell stock at a cell bank (K-Stem Cell, Seoul, Korea). Written informed consent for research use was provided by donors. Cryopreserved stem cells, stored in liquid nitrogen vapor, were then thawed and re-cultured in growth medium (RKCM; K-Stem Cell) in accordance with the injection schedule described in the Good Manufacturing Practice. Harvested stem cells were anonymized and transported to our facility under cold storage conditions. When cells were 80–90% confluent, they were subcultured and expanded in RKCM. The cell characteristics obtained were consistent with previous findings obtained using the same culture methods, which were compatible with MSC (data not shown).[18]

hAdMSC at passage 3 were seeded at 1 × 10^5^ cells in a 100 mm dish and then grown at 37°C to confluency. After serum deprivation, hAdMSC were exposed to a hypoxic (5% O_2_) chamber for 6 hours, then cultured for an additional 24 hours under normoxic conditions for collection of the hypoxia-induced secretome. Next, the conditioned medium was ultrafiltrated using a membrane with a 3-kDa molecular weight cut-off to remove small molecular weight components such as salts and amino acids using an Amicon Ultra-15 Centrifugal Filter Unit with an Ultracel-3 membrane (Millipore, Bedford, MA, USA). The high molecular weight fraction obtained from hypoxia-conditioned supernatants contained a high volume of proteins secreted from hAdMSC.

### hAdMSC secretome protein analysis

High molecular weight fraction samples from hAdMSC cultured under normoxic or hypoxic conditions were stored at -20°C until further protein analysis. Concentrations of multiple cytokines and growth factors including GM-CSF, IL-6, VEGF, IGF-1, and TGFβ1 were assessed using a Multiplex Human Cytokine Panel (xMAP; Luminex Corp. Austin, TX, USA) according to the manufacturer’s instructions. Three samples were measured in duplicate. A LUMINEX 200 instrument (Luminex Corporation, Austin, TX) was applied to run plates and generate quantitative data.

### Irradiation-induced salivary hypofunction animal model

Eight to nine-week-old female C3H mice weighing 18–22 g were purchased from the Research Model Producing Center (Orient Bio, Seongnam, Korea). Animals were maintained under conventional clean conditions and provided with standard laboratory chow and sterilized water *ad libitum*.

To induce IR-induced salivary dysfunction, animals were firmly fixed in a plastic mold and irradiated with a 4 MV X-ray from a linear accelerator (Mevatron MD, Siemens Medical Laboratories Inc., Germany) using a single dose of 15 Gy at a focus-to-skin distance of 100 cm. Animals were locally irradiated in the head and neck region, including the salivary gland, with bodies shielded from the radiation field. The dosage required to induce hyposalivation without compromising general health was calculated as previously described.[19]

### Systemic infusion of hypoxia-preconditioned hAdMSC secretome

The high molecular weight fraction of 500 μL from the hAdMSC secretome was administered to IR-induced salivary hypofunction mice via the tail vein immediately after IR on seven consecutive days (IR + SEC group). The control group received an equal volume (500 μL) of vehicle (PBS) only (IR + PBS group). Non-irradiated mice served as a normal control group (CON group). Each group consisted of 35 C3H mice. At 1, 2, 4, 8 or 16 weeks after IR, morphological, functional, and histological evaluations were performed using seven mice from each group at each time point.

### Morphological analyses

#### Gross appearance, body, and gland weights

Body weights were measured at 4 and 16 weeks after IR. After saliva was collected, the animals were humanely euthanized. The submandibular glands were then harvested and surrounding fat and connective tissues were removed. Next, the weights of both submandibular glands were measured before fixation in 10% neutral formalin buffer and embedding in paraffin.

#### Histological evaluation of structural changes in salivary glands

The sections (4 um) of formalin-fixed, paraffin-embedded tissues were cut and stained with hematoxylin-eosin (H&E). Periodic acid Schiff staining was performed by oxidizing paraffin removed sections in 1% periodic acid for 10 minutes, rinsing in distilled water, washing in tap water for 1 minute, and then rinsing in distilled water. Finally, the sections were incubated with Schiff’s reagent for 20 minutes, rinsed in distilled water, washed with tap water for 15 minutes, and counterstained with Mayer’s hematoxylin for 2 minutes.

### Immunohistochemical evaluation of cellular effects of hAdMSC secretome

Immunohistochemical analyses for AQP5 (1:200, Alomone Labs, Jerusalem, Israel), CD31 (1:50, Abcam, Cambridge, UK), α-SMA (1:500, Merck Millipore, Darmstadt, Germany), and c-Kit (1:200, Santa Cruz, CA, USA) were conducted using the streptavidin/biotin method (Invitrogen, Camarillo, CA, USA). Briefly, sections were incubated in a humid chamber with primary antibody at 4°C overnight. For negative controls, distilled water was used in place of the primary antibody. Samples were washed three times for 5 min each with PBS, then incubated with biotinylated IgG followed by streptavidin-peroxidase conjugate. After washing an additional three times for 5 min each with PBS, sections were incubated with DAB substrate containing diaminobenzidine to detect immunoreactivity and then with Mayer’s hematoxylin. Staining was viewed using a microscope (Olympus BX43, Olympus, Tokyo, Japan). Three random sections from each mouse were evaluated by a blinded researcher and the staining area was measured in pixels by a software program (Metamorph, Molecular Devices Corporation, Sunnyvale, CA, USA).

### Functional analyses

#### Salivary flow rates and lag times

Salivary secretory function was determined by measuring the salivary flow rates (SFR) and time to salivation (lag time) at 4 and 16 weeks after IR. Saliva was collected from the floor of the mouth using a micropipette for 10 min after stimulation by intraperitoneal injection with pilocarpine (2 mg/kg). Collected saliva was placed in pre-weighed 1.5 ml microcentrifuge tubes, after which the SFR (μL/min) was calculated by dividing the weight (mg) of all saliva obtained by the collection time (min) (saliva was assumed to have a specific gravity of 1 mg/mL). Salivary lag time (sec) was defined as the time from stimulation to the beginning of saliva secretion.

#### Assessment of protein contents in saliva

Saliva samples obtained at 2 and 4 weeks after IR were centrifuged at 6000 rpm for 15 min. Total supernatant protein concentrations were determined by bicinchoninic acid (BCA) assay (Pierce, Rockford, IL, USA), after which 1 μg of total saliva protein was separated on SDS-PAGE and transferred to an Immobilon nitrocellulose membrane (Millipore, Billerica, MA, USA). Next, the membrane was blocked with 5% skim milk/TBS for 1 hour at room temperature, then rinsed with wash buffer and incubated with mouse anti-amylase antibody (Santa Cruz Biotechnology, Santa Cruz, CA, USA) at a 1:5000 dilution. Following overnight incubation with shaking at 4°C, the membranes were rinsed with wash buffer three times for 5 minutes each. This was followed by incubation with a 1:5000 dilution of anti-mouse IgG horseradish peroxidase conjugate (Santa Cruz Biotechnology, Santa Cruz, CA, USA) for 1 hour at 37°C. Finally, samples were rinsed with wash buffer as above, after which detection was conducted using enhanced chemiluminescence (ECL) western detection reagents (Elpisbio, Daejeon, Korea). The bands of interest were detected using a luminescent image analyzer (4000r, Kodak, Rochester, NY, USA), and the results were quantified using a software program (Kodak).

Amylase activity of secreted saliva was determined using a Salivary α-Amylase Assay Kit (Salimetrics LLC, State College, PA, USA) with 2-chloro-p-nitrophenol linked with maltotriose as the chromogenic substrate according to the manufacturer’s instructions. The amount of α -amylase activity present in the sample is directly proportional to the increase in absorbance at 405 nm observed using a standard laboratory plate reader.

Saliva EGF was measured by enzyme-linked immunosorbent assay (ELISA) using a commercial kit (Quantikine; R&D Systems, Minneapolis, MN, USA) according to the manufacturer's instructions. The kit uses a sandwich ELISA that recognizes mouse EGF and has no detectable cross reactivity with other cytokines. Each sample from each group collected at different time points was assayed in duplicate and the plate was read at 450 nm. The EGF concentration of the samples was determined by reading the optical density of the sample against the values of the standard curve.

### Terminal deoxynucleotidyl transferase dUTP nick-end labeling (TUNEL) assay

Apoptotic cells in submandibular glands were visualized using an ApopTag Plus Fluorescein In Situ Apoptosis Detection kit (ApopTag^®^, Peroxidase *In Situ* Apoptosis Detection Kit, Millipore, Bedford, MA, USA), which uses terminal deoxynucleotidyl transferase dUTP nick-end labeling (TUNEL) to detect DNA cleavage and chromatin condensation. After deparaffinization and rehydration, slides were incubated with a TUNEL reaction mixture containing TdT enzyme for 1 h at 37°C, then with anti-digoxigenin fluorescein (peroxidase) for 30 min at room temperature. Nuclei were visualized using Mayer’s hematoxylin. A blinded examiner counted the TUNEL-positive cells in three random fields per tissue section. At least three random tissue sections per gland were mounted on each slide.

### Cell culture experiments

#### Preparation of human parotid gland epithelial cells

To evaluate the *in vitro* mechanism of the hAdMSC secretome, we cultured human parotid gland epithelial cells (HPEC) prepared using a specimen from a patient who underwent parotidectomy due to benign parotid tumor with informed consent and institutional IRB approval. Briefly, a small portion of a normal gland was resected and washed with cold DPBS containing 2% antibiotics three times. The tissue was then finely chopped with blades and enzymatically digested with 0.25% collagenase type B (2.5 mg/mL) and DNase I (1 mg/mL) with gentle shaking at 37°C for 30 minutes. The cell suspension was subsequently filtered through a 70 μm cell strainer and then centrifuged at 300 × g for 5 minutes, after which it was plated on a culture dish and cultured with keratinocyte-Serum Free Medium (KSFM; Gibco, Grand Island, NY, USA) containing 5 ng/ml epidermal growth factor, 0.09mM CaCl_2_ and 1% antibiotics.

HPEC were then examined to determine their salivary epithelial characteristics. Briefly, HPEC at passage 5 were seeded at 1 × 10^4^ cells/well, cultured at 37°C for 3 days, and then cultured on plates that had been precoated with Matrigel (BD Biosciences, Bedford, MA, USA) to induce 3-dimensional organization. The cells were subsequently photographed under a light microscope and prepared for RNA analysis, western blot, and immunofluorescence staining.

#### Cell proliferation and irradiation-induced cell death

HPEC were cultured on an 8-well slide chamber at a density of 2×10^4^ cells per well and then irradiated with 10 Gy using an IBL-437 irradiator (CIS Bio International, Nice, France), after which they were treated with hypoxia-conditioned hAdMSC medium at 10, 50, and 100% and a normoxic control medium for 72 hours. Apoptotic cells were visualized using the ApopTag^®^ Peroxidase *In Situ* Apoptosis Detection Kit as described above. A blinded examiner independently counted the numbers of apoptotic cells. Cell proliferation was assessed using a Cell Counting Kit-8 (CCK-8; Dojindo, Gaithersburg, MD, USA) in triplicate according to the manufacturer's instructions. Aliquots of HPEC (100 μL/8000 cells) cultured in 96-well plates were serum-starved overnight and then treated with 10, 50, or 100% conditioned medium or a normoxic control medium for 72 hours. The CCK-8 reagent (10 μL) was subsequently added to each well 1 h before completing the incubation. Finally, the absorbance at 450 nm was measured using a microplate reader.

#### Immunofluorescent staining

Anti-E-cad (R&D Systems, Minneapolis, MN), anti-ZO1 (Santa Cruz Biotechnology, Santa Cruz, CA, USA), and anti-AQP5 (Alomone Lab, Jerusalem, Israel) antibodies were used for immunofluorescence staining. After washing in PBS, cells were incubated with Alexa-488-conjugated goat anti-rabbit IgG for 2 h in the dark at room temperature. Next, 4’,6-diamidino-2-phenylindole, dihydrochloride (DAPI; Vector Labs, Burlingame, CA) was added for 3–5 minutes to stain the cell nuclei. All experiments included a slide with no primary antibody as a negative control. After mounting, cells were viewed using a confocal laser scanning microscope (Olympus FV1000, Olympus, Tokyo, Japan).

#### Quantitative real-time-PCR analysis

The levels of transcripts were determined by real-time PCR (RT-PCR) on the ABI PRISM sequence detection system using SYBR Green I as a double-stranded DNA-specific dye according to the manufacturer’s instructions (Applied Biosystems, Foster City, CA, USA). The PCR reaction was carried out using 1μM of cDNA, 10μM of SYBR Green PCR master mix, 10pM of sense and antisense primer of AQP5: (forward 5’-ACT GGG TTT TCT GGG TAG GG-3’, reverse 5’-GTG GTC AGC TCC ATG GTC TT-3’), CK7: (forward 5’-CAG GAT GTG GTG GAG GAC-3’, reverse 5’-AAC TTG GCA CGC TGG TTC T-3’) in a final volume of 20 μL per reaction. The amount of real-time PCR products was normalized against the house-keeping gene, β-actin.

#### Western blot

Protein samples were isolated from the lysate (50 μg) and mixed in reducing buffer, boiled, resolved on SDS-PAGE gels, and transferred to a polyvinylidene difluoride membrane (PVDF) by blotting. The blot was then incubated overnight at 4°C in a blocking solution with primary antibodies against ZO-1, CK7, β-actin (Santa Cruz Biotechnology, CA, USA), BAX, Bcl-2 and cleaved Caspase-3 (Cell Signaling, Danvers, MA, USA). After washing the blots with 0.1% Tween 20 in PBS, they were incubated with horseradish peroxidase-conjugated secondary antibodies corresponding to each primary antibody and then subjected to enhanced chemiluminescence detection (GE Healthcare Life Science, Piscataway, NJ, USA).

### Statistical analysis

Statistical analysis was conducted using the Graph Pad Prism 5 package (GraphPad Software Inc., La Jolla, CA, USA). The Mann-Whitney test was used to identify differences between groups, and one way ANOVA followed by Tukey’s post hoc test and two-way ANOVA followed by the Bonferroni post hoc test were used to compare values among groups. Linear regression was applied to evaluate the correlation between parameters. A p < 0.05 was considered to indicate statistical significance.

## Results

### Concentration of hAdMSC-secreted cytokines and growth factors

**Fig 1:**
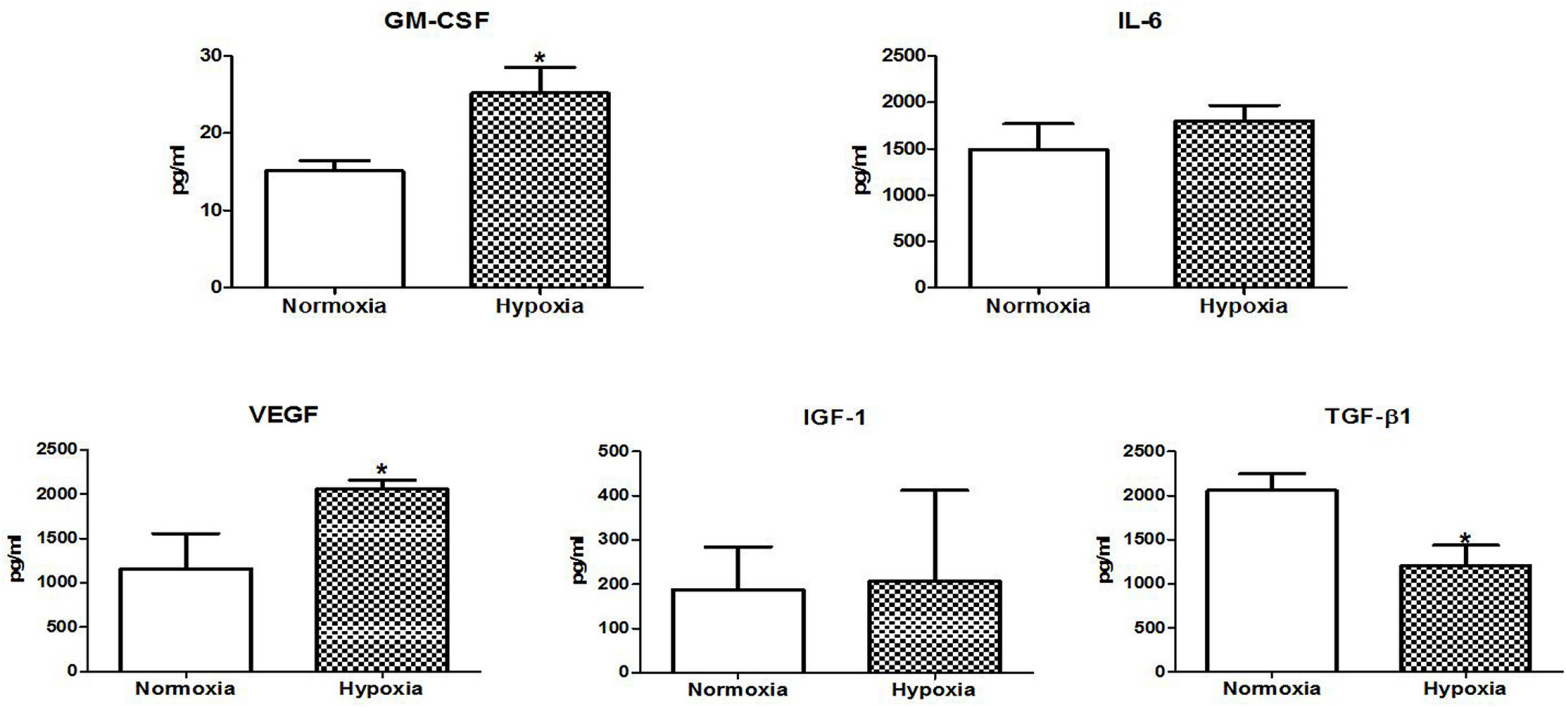
Secretome analysis. Concentrations of cytokine and growth factors released by hAdMSC were measured after hypoxia preconditioning with O_2_ at < 5% for 6 hours using multiplex antibody arrays. Data displayed are from the hAdMSC secretomes generated under normoxia and hypoxia. Mann-Whitney test (A) GM-CSF, P = 0.048, (B) IL-6, P = 0.3914, (C) VEGF, P = 0.044, (D) IGF-1, P = 0.936; n = 3. Each sample was measured in duplicate.

We initially analyzed the concentrations of hAdMSC-secreted therapeutic factors released under hypoxic culture conditions and compared them with those from normoxic culture and standard culture medium using multiplex antibody arrays. The concentrations of several cytokines and growth factors were altered in the hypoxia-conditioned cultures relative to the normoxia cultures (Table 1 and Fig 1). In addition, hypoxia led to significantly more GM-CSF (median 25.18 pg/ml, P = 0.148) and VEGF (median 2054.33 pg/ml, P = 0.044) production in the hAdMSC secretome than proteins in normoxia (median 13.95 pg/ml and 788 pg/ml, respectively). Although IL-6 and IGF-1 did not increase significantly, they showed a more than 1.5 fold increase. TGF-β1 levels were significantly lower in the hypoxia preconditioned secretome than the one generated under normoxia (1975 pg/ml versus 1204 pg/ml, P = 0.048). The amounts of these proteins in the FBS 5% control medium were significantly lower than in the hAdMSC cultured medium.

**Table 1:** Concentration of hAdMSC-derived secretome.

Cytokines (pg/ml)	Control medium FBS 5%	hAdMSC secretome, Normoxia, median (range)	hAdMSC secretome, Hypoxia, median (range)	Fold changes	P
GM-CSF	0.17	13.95 (13.63–17.74)	25.18 (20.54–31.64)	1.8	0.048
IL-6	0.3	1371 (1073–2020)	1800.33 (1535–2107)	1.5	0.391
VEGF	0	788 (733–1950)	2054.33 (1855–3295)	2.6	0.044
IGF-1	59	229 (0–331)	619 (0–619)	2.7	0.936
TGF-β1	9.12	1975 (1768–2421)	1204 (752–1524)	0.6	0.048

hAdMSC: human adipose-derived mesenchymal stem cells.

### Macro- and micro-morphological improvement after treatment with the hAdMSC-derived secretome

**Fig 2:**
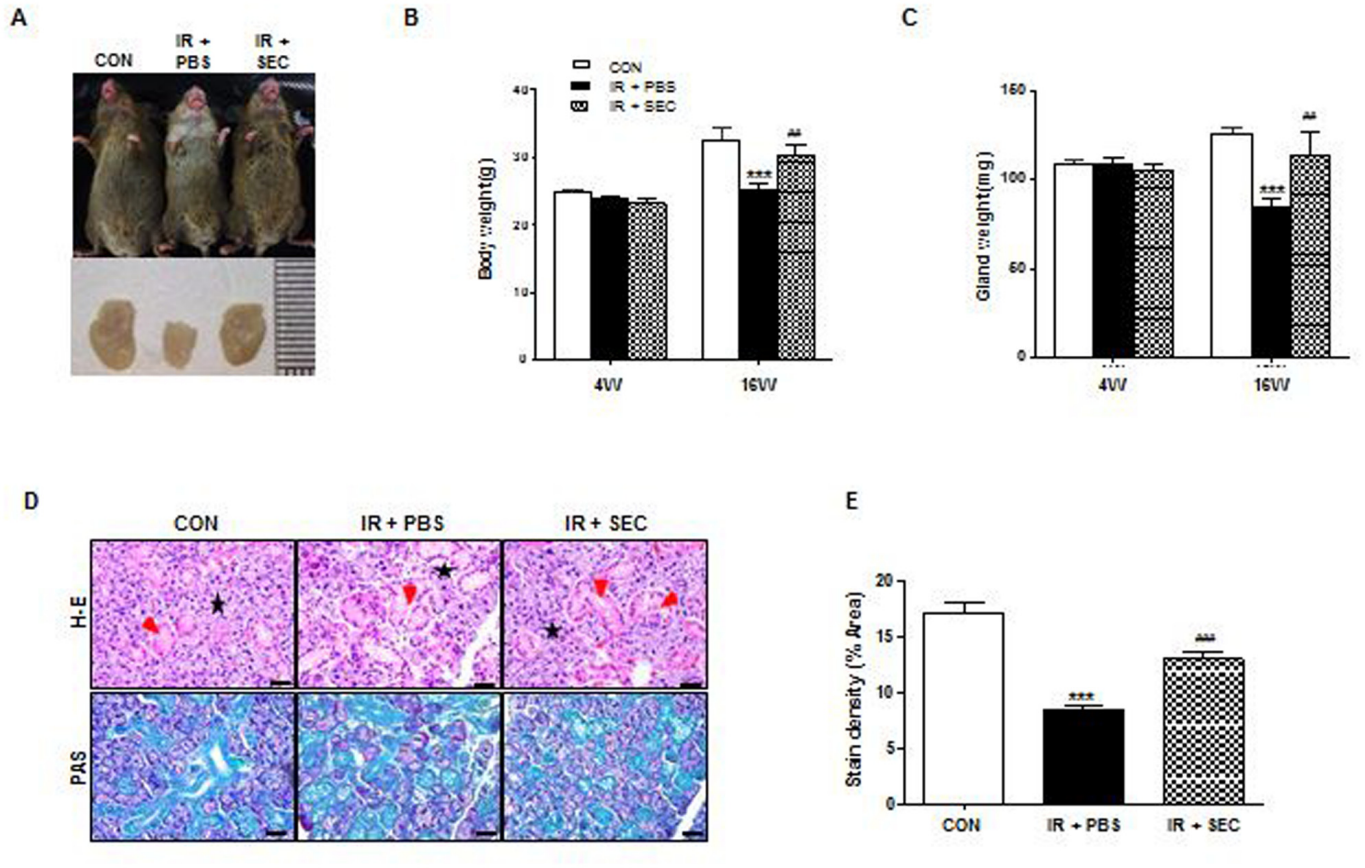
Macro- and micro-morphological evaluation. (A) External appearance and dissected SGs of mice in each group were photographed at 16 weeks after IR. (B and C) Body weight and dissected glandular weight were measured at 4 and 16 weeks after IR. (D) Representative histological pictures of H-E staining (upper) and PAS staining (lower) from three groups at 16 weeks post-IR are presented. Red arrows indicate ductal structures and stars mark acinar structures. Scale bars represent 50 μm. (E) Densities upon PAS staining were measured using a software program to calculate pixels of purple stained mucin-containing areas. Data are presented as the mean ± SEM. Two-way ANOVA, Bonferroni post hoc test (B and C), One-way ANOVA, Tukey’s pot hoc test (E). *, compared to CON; ^#^, compared to IR + PBS. ***P <0.001, ^##^P < 0.01, and ^###^P < 0.001. CON, normal control group (n = 6–7); IR + PBS, PBS-treated group (n = 6); IR + SEC, secretome-treated group (n = 6).

External appearance and dissected salivary glands of mice in each group were observed at 16 weeks after IR (Fig 2A). Neck IR resulted in loss of hair around the neck and decreased glandular size, but hAdMSC secretome-treated mice showed less hair loss and larger glandular size than the IR + PBS group, which was comparable with the control group.

Body weight and dissected glandular weight were measured at 4 and 16 weeks after IR (Fig 2B and 2C). Body weight and glandular weight were significantly decreased in the PBS group relative to the control group at 16 weeks after IR (P < 0.001). However, hAdMSC secretome treatment exhibited significantly increased body and glandular weight compared to the PBS group (P < 0.01).

Histological evaluations of the micromorphological changes in the three groups were performed and the densities of mucin were measured by PAS staining followed by examination by a blinded researcher using a software program to calculate the pixels of purple stained areas at 16 weeks after IR. The salivary acinoductal structure was found to be destroyed and reduced after IR, but were maintained relatively well in the hAdMSC secretome group (Fig 2D). Morphometric analysis of PAS staining showed that the production of mucin decreased significantly after IR (P <0.001), but increased significantly in the hAdMSC secretome group relative to the PBS group (Fig 2E, P < 0.001).

### Cytoprotective effects of hAdMSC treatment

**Fig 3:**
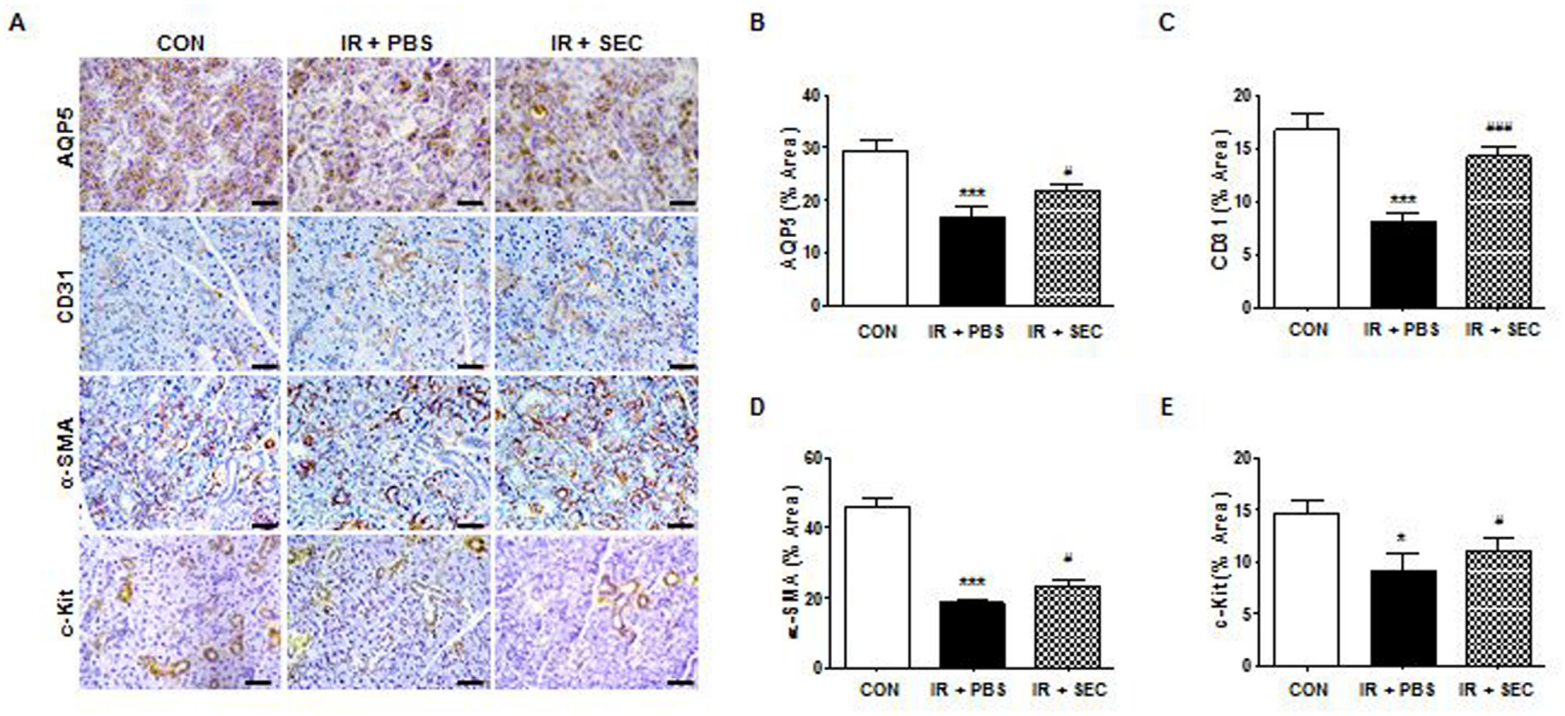
Cytoprotective effect of hAdMSC secretome treatment. (A) Representative images of salivary epithelial (AQP5), endothelial (CD31), myoepithelial (α-SMA), and progenitor cells (c-Kit) in the SG in the three experimental groups. Scale bars represent 50 μm. (B-E) Each staining area was measured in pixels using a software program. Data are presented as the mean area (%) ± SEM. One-way ANOVA, Tukey’s pot hoc test. *, compared to CON; ^#^, compared to IR + PBS. *P < 0.05, ***P <0.001, ^#^P < 0.05, and ^###^P < 0.001. CON, normal control group; IR + PBS, PBS-treated group; IR + SEC, secretome-treated group. Three random sections from each mouse were evaluated by a blinded researcher. The total number of slides examined from each experimental group ranged from 18 to 21.

We next investigated the cellular effects of infusion of the hAdMSC secretome on salivary epithelial, endothelial, myoepithelial, and progenitor cells by detection of immunohistochemical markers (Fig 3A). Immunostaining revealed that expression of AQP5 (a marker of salivary epithelial cells), CD31 (endothelial cells), α-SMA (myoepithelial cells), and c-Kit (progenitor cells) was significantly lower in the PBS group than the control group (Fig 3B–3E, P < 0.001 in epithelial, endothelial, and myoepithelial cells, and P < 0.05 in progenitor cells). Administration of the hAdMSC secretome increased the staining density of these cells relative to the PBS group (P < 0.001 in endothelial cells and P < 0.05 in epithelial, myoepithelial, and progenitor cells).

### Recovery of salivary hypofunction by hAdMSC secretome infusion

**Fig 4:**
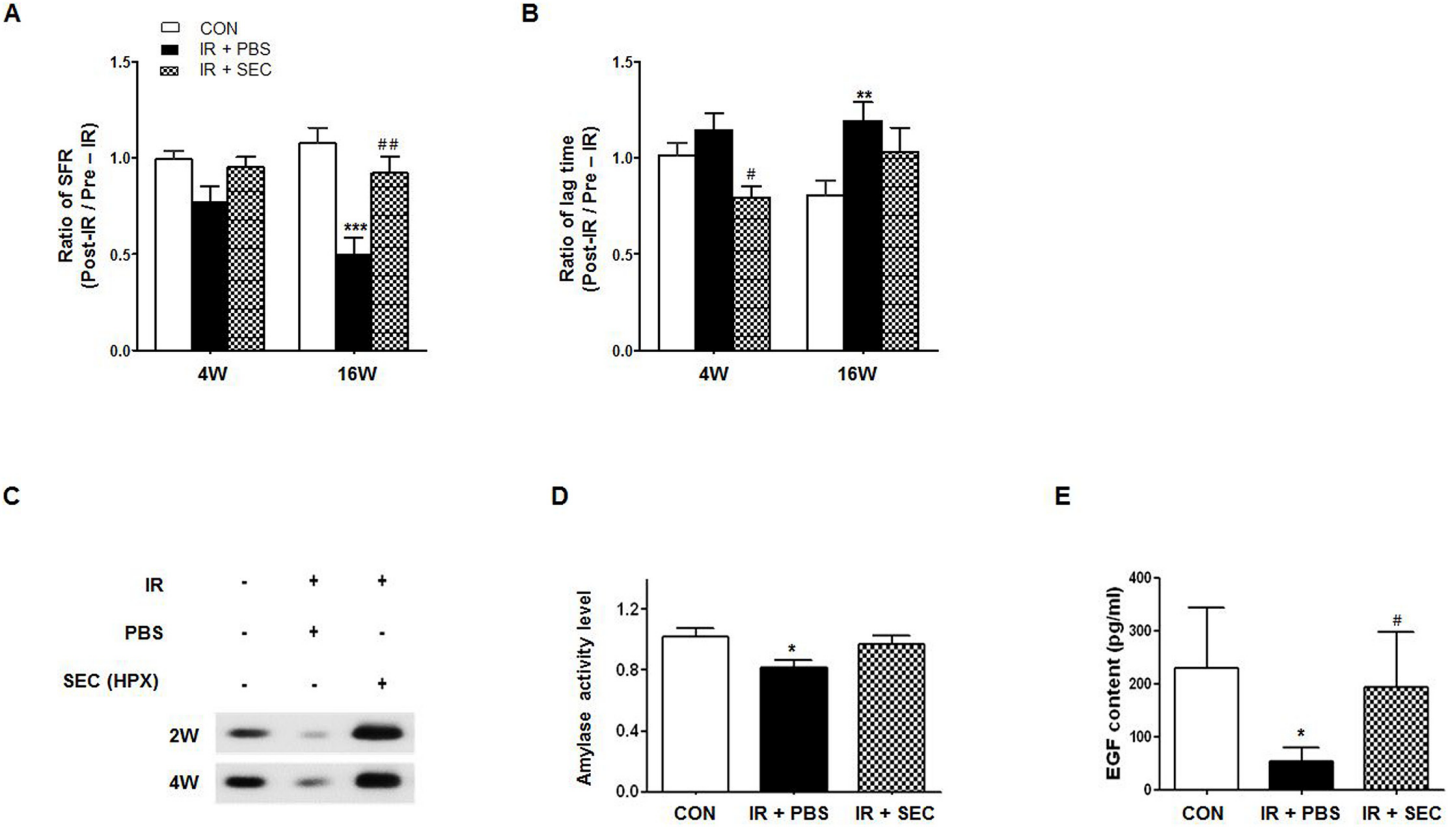
Recovery of salivary hypofunction by hAdMSC secretome infusion. (A) Salivary flow rate (SFR) was calculated at pre-IR and 4 and 16 weeks post-IR. The changes in SFR after IR were expressed by the ratio of post-IR SFR to pre-IR SFR (Mean ± SEM). (B) Time to salivation (lag time, LT) was measured and the ratios of post-IR LT to pre-IR LT were presented. (C) Western blotting of amylase in saliva at 2 and 4 weeks post-IR. (D) The salivary amylase activity was examined by the Assay Kit and fold changes in activity level are presented. (E) EGF contents were measured at each time point and the average contents are presented. Data are presented as the mean ± SEM. Two-way ANOVA, Bonferroni post hoc test (A and B), one-way ANOVA, Tukey’s pot hoc test (D and E). *, compared to CON; ^#^, compared to IR + PBS. *P <0.05, **P < .01, ***P <0.001, ^#^P < 0.05, and ^##^P < 0.01. CON, normal control group (n = 5–7); IR + PBS, PBS-treated group (n = 5–6); IR + SEC, secretome-treated group (n = 5–6).

The amount of salivation and lag time were measured pre-IR and 4 and 16 weeks post-IR (Fig 4A and 4B). The changes in SFR and lag time after IR were calculated by the post-IR to pre-IR ratio. IR significantly decreased the ratio of SFR and increased the ratio of the lag time at 16 weeks after IR (Fig 4A, P < 0.001 and P < 0.01, respectively). hAdMSC infusion significantly improved the ratio of SFR at 16 weeks after IR (Fig 4A, P < 0.01) and the lag time at 4 weeks after IR relative to the PBS group (Fig 4B, P < 0.05).

We performed Western blotting of saliva collected at 2 and 4weeks post-IR to examine the salivary amylase protein expression level (Fig 4C). The expression levels of salivary amylase were higher after infusion of the hAdMSC secretome relative to the PBS group. The average activity of salivary amylase was significantly reduced in the PBS group (Fig 4D, P < 0.01) although it was not significantly improved in the secretome group. EGF contents were also measured at each time point. The average contents were significantly lower in the PBS group than the control group (Fig 4E, P<0.05), and the average EGF content was significantly increased in the hAdMSC secretome group relative to the PBS group (P < 0.05).

### The role of hAdMSC secretome in salivary gland tissue remodeling

hAdMSC secretome exerts anti-apoptotic effects on SGs in vivo and in vitro.

**Fig 5:**
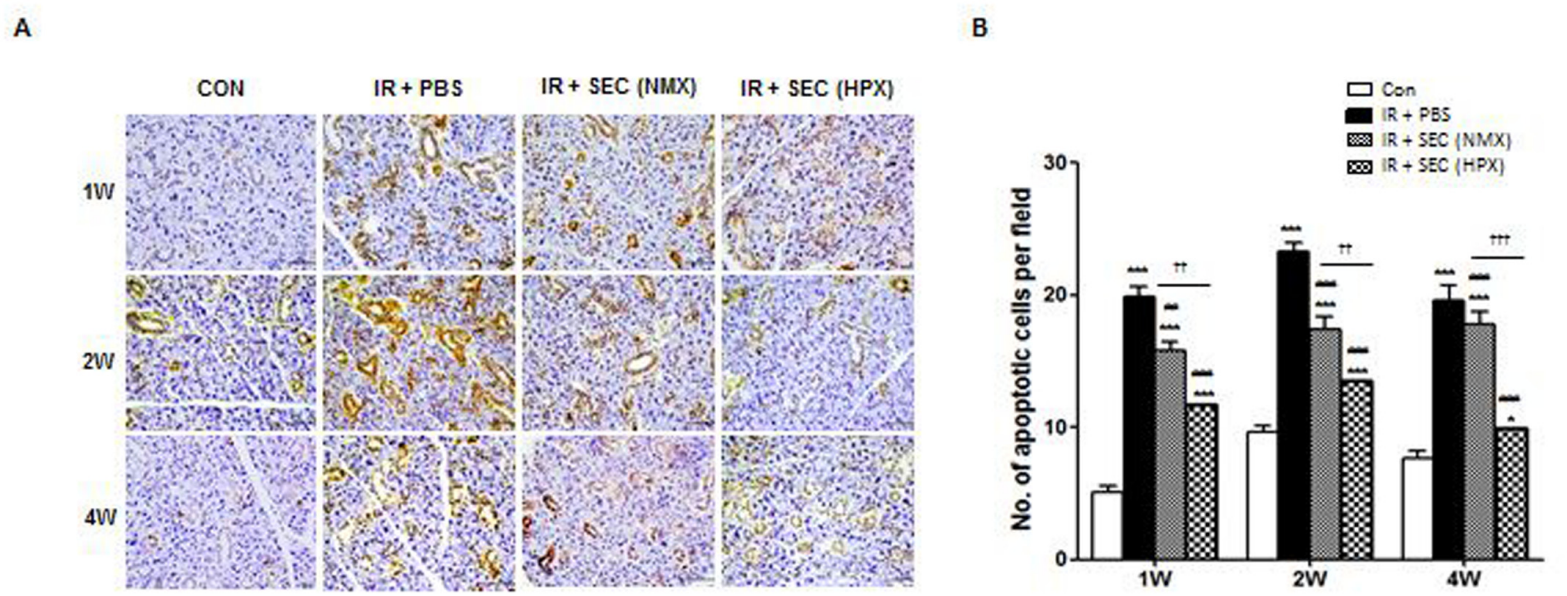
hAdMSC secretome exerts cytoprotection via anti-apoptotic effects on salivary parenchymal cells *in vivo*. (A) Representative images of an *in vivo* TUNEL assay from three experimental groups at 1, 2, and 4 weeks post-IR. Scale bars represent 50 μm. (B) The number of TUNEL-positive apoptotic cells was determined by a blinded researcher. Data are presented as the mean number of apoptotic cells per field ± SEM. Two-way ANOVA, Bonferroni post hoc test. *, compared to CON; #, compared to IR + PBS; †, compared to IR + NMX. *P <0.01, ***P <0.001, ^##^P < 0.01, ^###^P < 0.001, ††P<0.01, †††P<0.001. CON, normal control group (n = 21 sections); IR+ PBS, PBS-treated group (n = 21 sections); IR + SEC (NMX), IR + normoxic secretome-treated group (n = 15 sections); IR + SEC (HPX), hypoxia-conditioned secretome-treated group (n = 18 sections).

We next explored whether these cytoprotective effects of the hAdMSC secretome were related to inhibition of cell death by MSC-secreted paracrine factors. The number of TUNEL-positive apoptotic cells after infusion of PBS, normoxia-cultured hAdMSC secretome, or hypoxia-preconditioned hAdMSC secretome was determined at 1, 2, and 4 weeks post-IR (Fig 5A). A significant increase in apoptosis was observed after IR during the observation period (Fig 5B, P < 0.001), but this decreased significantly following hAdMSC secretome treatment relative to the PBS group (P < 0.01 at 1 week in the normoxia-secretome group and P < 0.001 at 2 and 4 weeks in the normoxia-secretome and all time points in the hypoxia-secretome). Treatment with the hypoxia-conditioned hAdMSC secretome resulted in enhanced anti-apoptotic effects relative to the normoxia-secretome (P < 0.01 at 1 and 2 weeks and P < 0.001 at 4 weeks after treatment).

**Fig 6:**
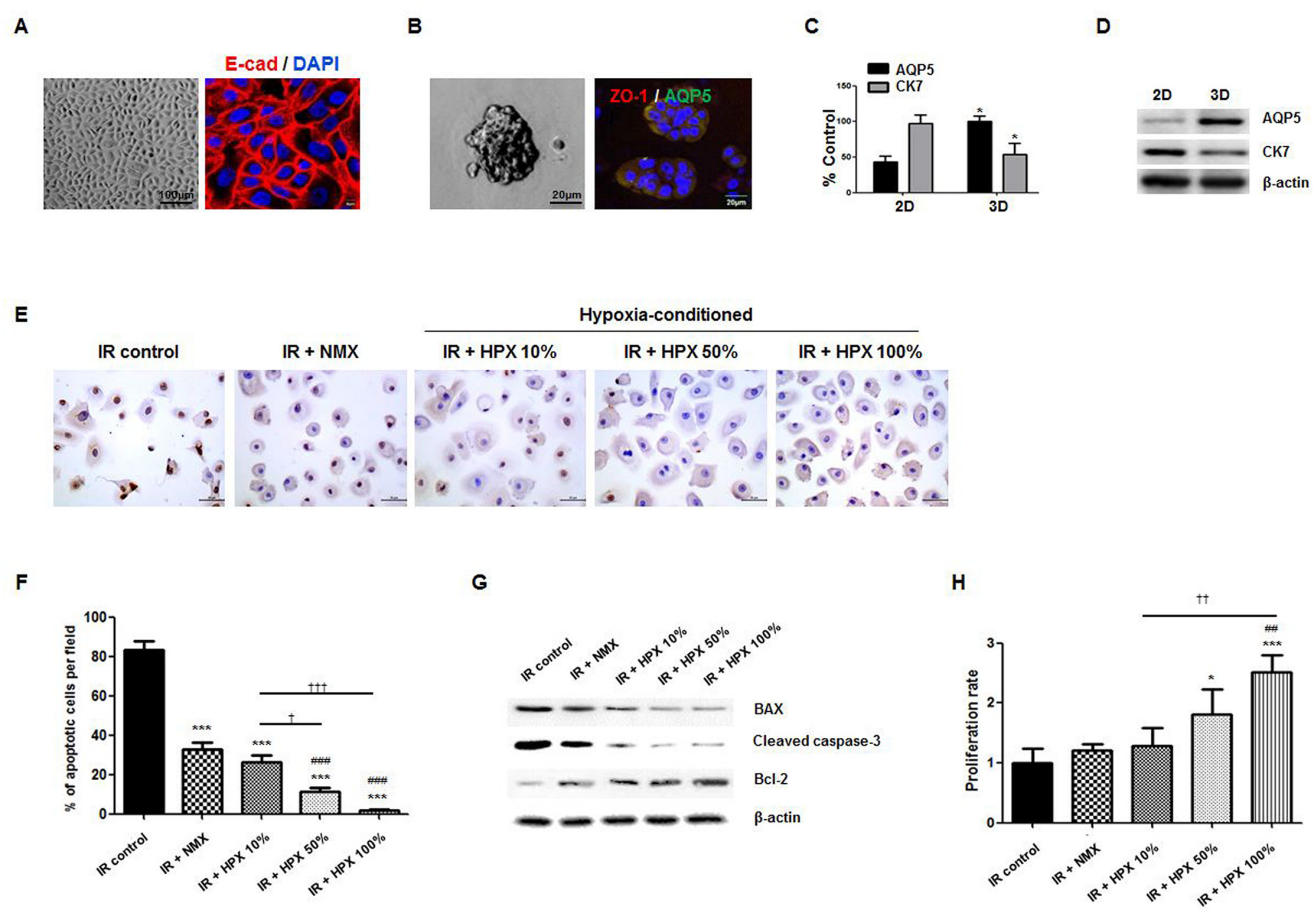
*In vitro* effect of the hAdMSC secretome on cell death and proliferation of human parotid epithelial cells (HPEC) treated with the hAdMSC secretome at concentrations of 10, 50 and 100% or a normoxic control medium. (A–B) Light microscopic and immunofluorescent staining of HPEC. Scale bars represent 100 and 8μm in A and 100 and 20 μm in B. (C-D) Salivary epithelial genes of AQP5 and CK7 were confirmed by real-time PCR and western blot. *, compared to 2D, *P < 0.05. (E) Anti-apoptotic effect was confirmed by an *in vitro* TUNEL assay in HPEC. (F) The number of fragmented DNA of HPEC according to the concentration of hAdMSC-cultured medium was determined in the same manner. Data are presented as the mean number of apoptotic cells per field ± SEM. One-way ANOVA, Tukey’s pot hoc test. *, compared to 0%; #, compared to IR + PBS; †, compared to IR + NMX. ***P < 0.001, ^###^P < 0.001, †P < 0.05, †††P < 0.001. 0%, control standard medium. (G) IR-induced changes in Bax, cleaved caspase-3 and Bcl-2 in HPEC treated with the hypoxia-cultured hAdMSC medium at concentrations of 10, 50 and 100% or a normoxic control medium for 72 hours were evaluated using Western blot. (H) Cell proliferation of HPEC was assessed in triplicate using a CCK-8. Data are presented as the mean number of cells per field ± SEM. One-way ANOVA, Tukey’s pot hoc test. *, compared to 0%; #, compared to IR + PBS; †, compared to IR + NMX. *P < 0.05, ***P < 0.001, ^##^P < 0.01, ††P < 0.01; IR + NMX, IR + normoxia-cultured hAdMSC medium, IR + HPX, hypoxia-conditioned hAdMSC medium.

To confirm the anti-apoptotic effects and evaluate its dose-response relationship in an *in vitro* cell culture model, we isolated primary cells from human parotid glands. The characteristics of salivary epithelial cells were then confirmed based on exhibition of a polygonal shape upon 2D culture (Fig 6A), and acinar-like 3D spheroid organization on Matrigel (Fib 6B). The cells were further confirmed to express salivary epithelial markers such as E-cadherin on 2D culture and AQP-5 and ZO-1 on 3D culture (Fig 6A and 6B). Salivary epithelial genes of AQP-5 and CK7 were also confirmed by PCR and western blot (Fig 6C and 6D), and the acinar-like structure showed increased expression of acinar marker of APQ-5 when cultured on 3D Matrigel.

We then performed an *in vitro* TUNEL assay, which revealed the presence of fragmented DNA of HPEC. These findings are direct evidence of apoptotic cell death. The fragmented DNA decreased significantly following treatment of the secretome, where HPEC cultured in the hypoxia-conditioned medium showed less cell death relative to that cultured in the normoxic medium (Fig 6E and 6F). The anti-apoptotic effect of hypoxia-conditioned hAdMSC medium was significantly enhanced in a dose-dependent fashion, and HPEC cultured in 50% and 100% hypoxic medium exhibited a significant reduction of cell death relative to 10% of concentration of hypoxic medium (Fig 6F, P < 0.05 in 50% and P < 0.001 in 100% of concentration). We also evaluated the effects of hAdMSC secretome on apoptosis by determining the changes in apoptosis-associated markers induced by IR. Treatment with hypoxic medium blocked the IR-induced alteration of expression of Bax, cleaved caspase-3, and Bcl-2 in HPEC (Fig 6G). These results demonstrate that the therapeutic effects of the hAdMSC secretome could be enhanced by hypoxia-preconditioning. In addition, HPEC showed significant increases in proliferation in response to treatment with increasing concentrations of hypoxia-conditioned medium relative to normoxic control medium treatment (Fig 6H, P > 0.01 in 100% hypoxic medium relative to normoxic medium and 10% hypoxic medium).

## Discussion

Local paracrine effects have been proposed as the main mechanism of SG tissue remodeling. Although soluble intracellular contents lysed from bone marrow cells, even from those in an inactive state, contribute to the repair and regeneration of SGs damaged by IR,[20] it is important to realize that these paracrine functions must be optimized by modulation of MSC to enable clinical translation. In this study, we modulated MSC by subjecting samples to hypoxic conditions to enhance paracrine function, then prepared a high molecular weight fraction. Systemic infusion of the hypoxia-preconditioned hAdMSC secretome successfully restored SG function against IR-induced damage. Our results revealed that the cell-free MSC secretome had a therapeutic benefit on remodeling of IR-damaged SG tissue via cellular protection. These results also support the belief that MSC preconditioning by subjection to hypoxia could promote the regeneration of IR-inflicted tissue following irradiation, and that MSC paracrine signaling could address an important mechanism of culture-expanded MSC therapy for IR-induced SG disorders.

It has been suggested that hypoxia preconditioning stimulates MSC to secrete paracrine factors to enable cellular survival in hypoxic and harsh microenvironments. For example, several angiogenic or antiapoptotic cytokines including VEGF, bFGF, and HGF are considered to be upregulated in MSC under hypoxia.[21,22] Gao et al. demonstrated that repeated infusion of hypoxia-preconditioned MSC-derived trophic factors after abdominal IR improved intestinal tissue homeostasis.[23] In the present study, we analyzed protein levels of the secretome released from hypoxia-preconditioned hAdMSC and found that GM-CSF, IL-6, VEGF, and IGF-1 increased and TGF-β1 decreased relative to normoxia-conditioned medium. Although it is unclear whether secretion varies according to exposure time and degree of hypoxia, this physiological conditioning could contribute to stimulation of MSC paracrine secretion, especially those associated with tissue remodeling and angiogenesis.

Our study demonstrated an ability of the hAdMSC secretome to repair and remodel SG structures, thus restoring the SG function following IR. After repeated hAdMSC secretome infusion, SG acinoductal structural integrity was maintained relative to IR-induced acinar cell losses and the resulting reduced mucin production. SGs acutely respond to IR, although salivary gland epithelial cells have slow turn-over. Salivary flow abruptly decreased to 50–60% in the first week after IR, then gradually decreased to less than 10% of the initial flow.[24,25] Furthermore, salivary proteins including mucin and EGF, electrolytes, and antibacterial systems can be altered.[26] We measured both quantitative (salivation capacity) and qualitative (salivary proteins) salivary function and found functional rescue after systemic MSC secretome infusion. Our data are consistent with previous reports that soluble intracellular contents lysed from bone marrow cells successfully restored salivary function,[20] which supports the finding that MSC paracrine factors provide an opportunity to restore IR-induced SG hypofunction without cell transplantation.

In this study, more c-Kit expressing SG progenitor cells were found in MSC secretome-treated SGs than untreated IR controls, indicating that the contribution of the MSC secretome to proliferation of SG progenitor cells and reduction of cell death after IR exposure may address an important *in vivo* mechanism of the MSC secretome. Since MSC treatment could preserve tissue-specific stem/progenitor cells with the ability to continuously expand and self-organize epithelial structure after radiation exposure, partial survival of SG progenitor cells is likely to induce remodeling of IR-damaged SGs.

In addition to failure to replenish resident stem cells, loss of acute responding parenchymal cells is another important mechanism of IR-induced tissue damage. We observed that SG parenchymal cells could be rescued by the hAdMSC secretome *in vivo* and *in vitro*. The hAdMSC secretome exerted its cytoprotective effects through promotion of cell proliferation and reduction of apoptosis after irradiation. Moreover, the anti-apoptotic effects of the hAdMSC secretome were found to be promoted after hypoxia-preconditioning relative to normoxia-cultured hAdMSC secretome. We attribute this cellular preservation to the direct effects of trophic factors; however, further investigation is still necessary to determine which potent factors released from MSC in the glandular microenvironment *in vivo* following damage due to radiotherapy account for SG remodeling and functional rescue. Elucidation of the molecular pathway mediating the effects of the MSC secretome is the next step toward enabling clinical utility of cell-free MSC secretome.

The main mechanism of the effects of MSC on tissue repair and regeneration is through paracrine activity that produces multiple trophic factors with various properties. Indeed, MSC release many trophic factors in injured microenvironments, which can attenuate tissue damage by inhibiting apoptosis, inflammation, and fibrotic remodeling, as well as promote angiogenesis and endogenous stem cell recruitment.[27] The MSC secretome would have certain advantages over injection of living MSC or administration of a single therapeutic factor or a combination of factors.[28] However, further research regarding the MSC secretome has been limited by their heterogeneity according to diverse culture conditions. Recently, extracellular vesicles (exosomes and microvesicles) have been shown to play a major role in the reparative effects of MSC, and many efforts have been made to purify them through ultracentrifugation or mirco-filters.[29] Since the therapeutic effects of extracellular vesicles have not yet been addressed in IR-induced SG damage, further comparative studies are still needed.

It should be noted that this study has several limitations. First, we used a single dose of 15 Gy in our animal model. Although this is sufficient to induce SG hypofunction in a mouse model without compromising general health, a fractionation-induced hypofunction model would be more relevant to clinical settings for future clinical translation. Second, we started to systemically infuse the hAdMSC secretome immediately after IR and repeated treatment for 7 consecutive days; however, we are not certain that this treatment could also exert beneficial effects on established IR wounds. If whole tissue stem/progenitor cells are chronically depleted, the therapeutic effects of this treatment may be minimal. Future studies should be conducted to determine the optimal timing and delivery route of therapeutic drugs for patients who are supposed to receive IR. Finally, although we can postulate that this cell free strategy is attractive for future clinical applications, it is clear that further work is necessary to profile and optimize formulations of the MSC secretome. Accordingly, a complete list of constitutively expressed MSC paracrine factors should be generated. Additionally, work should be continued to identify hypoxia-specific key factors secreted from hypoxia-preconditioned MSC and elucidate the mechanisms through which they show enhanced effects on SG tissue repair rather than those of normoxic MSC secretome. However, to the best of our knowledge, no novel paracrine factors released from MSC for salivary gland regeneration have been identified to date. Clearly, safety concerns and sustainability should be confirmed in future studies.

In conclusion, this study provides evidence of the potential for the hAdMSC secretome to remodel IR-damaged tissue and restore IR-induced SG hypofunction through cytoprotection of SG parenchymal, endothelial and progenitor cells by inhibition of apoptosis, or renewal of SG cells from SG tissue-resident stem/progenitor cells. Hypoxia-preconditioning of MSC could enhance trophic effects and secrete cytokines or growth factors, which involves tissue remodeling and angiogenesis. Administration of these therapeutic drugs, including multiple cytokine/growth factors without cell transplantation, could provide an opportunity for tissue regeneration in IR-damaged SGs.
